# Host stress proteins shape hemorrhagic shock via gut microbiota: evidence from Mendelian randomization and animal models

**DOI:** 10.1186/s12967-025-07364-8

**Published:** 2025-11-20

**Authors:** Gaorong Deng, Liping Wu, Shui Xiong, Junxin Zhou, Zongfang Li

**Affiliations:** 1 Department of Orthopaedics, Second Affiliated Hospital of Xi’an Jiaotong University, No. 157, West Fifth Road, Xincheng District, Xi’an City, 710004 China; 2 College of Life Science, Jiangxi Science and Technology Normal University, Nanchang, China; 3 Nanchang University Affiliated Rehabilitation Hospital Orthopedics Department, Rehabilitation Hospital Affiliated to Nanchang University, No. 133, South Square Road, Nanchang City, Jiangxi China; 4 National-Local Joint Engineering Research Center of Biodiagnostics and Biotherapy, Second Affiliated Hospital of Xi’an Jiaotong University, No. 157, West Fifth Road, Xincheng District, Xi’an City, 710004 China

**Keywords:** Hemorrhagic shock, Gut microbiota, 16S rRNA sequencing, Heat shock proteins, Apoptosis, Hypoxia-inducible factors, Biomarkers, Probiotics, Short-chain fatty acids (SCFAs)

## Abstract

**Background:**

Hemorrhagic shock (HS) is a severe condition involving stress proteins, inflammation, and gut microbiota dysbiosis. Understanding whether regulatory proteins influence HS through microbial pathways is crucial for improving therapeutic strategies.

**Methods:**

We used Mendelian randomization (MR) combined with animal experiments to investigate the role of regulatory proteins in HS. Two-sample MR was performed to assess the impact of various stress-related proteins. Additionally, 16 S rRNA sequencing was conducted in a rat HS model to analyze gut microbiota diversity and composition at baseline, 24 h, and 72 h after hemorrhage.

**Results:**

Two-sample MR identified HSPB1 and HIF1A as protective proteins, while APAF1, F7, and F10 increased susceptibility to HS. In the rat model, microbiota alpha diversity decreased at 24 h but partially recovered by 72 h, with significant shifts in beta diversity. Genus-level analysis revealed transient expansion of Lactobacillus, followed by dominance of Blautia and Romboutsia. Stage-specific predictions from PICRUSt2 suggested enrichment of amino acid metabolism and protein synthesis, particularly at 72 h, implicating microbial regulation in cellular recovery and stress adaptation.

**Conclusions:**

Our findings support a “protein-microbiota-HS” regulatory framework, highlighting the gut microbiota as key mediators of host stress responses. This integrative approach provides mechanistic insights into HS pathogenesis and suggests potential microbiome-targeted therapeutic strategies. We propose that targeting specific microbial communities, such as Blautia and Lactobacillus, could enhance recovery from HS.

**Supplementary Information:**

The online version contains supplementary material available at 10.1186/s12967-025-07364-8.

## Introduction

Hemorrhagic shock (HS), a life-threatening condition triggered by acute blood loss, is a common and severe complication of trauma, major surgery, and various critical illnesses [1–3]. Clinically, HS is characterized by its rapid onset, swift progression, and high mortality [4, 5]. The underlying pathophysiology involves a sudden reduction in circulating blood volume, leading to tissue hypoperfusion, metabolic acidosis, and systemic hypoxia, ultimately precipitating multi-organ injury [6–10]. Beyond the initial insult, HS is a leading cause of multiple organ dysfunction syndrome (MODS) and death in critically ill patients [11, 12].

The progression of HS is marked by a complex network of pathophysiological responses, including immune hyperactivation, cytokine storm, coagulation dysregulation, endothelial barrier disruption, and increased gut permeability accompanied by microbiota dysbiosis [13–16]. These interrelated processes form a self-amplifying cascade that exacerbates tissue damage [17]. Despite advances in fluid resuscitation, blood transfusion, mechanical support, and anti-inflammatory therapies, effective strategies for precisely targeting the core molecular mechanisms of HS remain lacking [18–21]. The temporal and spatial heterogeneity of HS pathogenesis, along with its multifactorial nature, poses significant challenges for mechanistic elucidation and therapeutic intervention [22, 23].

At the cellular level, the host rapidly mobilizes a repertoire of endogenous stress-response proteins in response to hypoxia, oxidative stress, and energy depletion [24]. Among these, heat shock proteins (HSPs) play a pivotal role in maintaining protein homeostasis, preventing aggregation, and regulating apoptosis [25]. Small HSPs such as HSPB1 are known to participate in autophagy, oxidative defense, and cytoskeletal remodeling, and may confer organ-protective effects in ischemia/reperfusion and shock models by modulating hypoxia and inflammation pathways [26–30]. Programmed cell death (apoptosis), a hallmark early event in HS, is driven by key regulators such as APAF1, BAX, and caspases, which are activated under energy stress and mitochondrial dysfunction, contributing to tissue damage and systemic inflammation [31–33].

In hypoxic environments, hypoxia-inducible factors (HIFs) orchestrate cellular adaptation by regulating genes involved in glycolysis, erythropoiesis, and angiogenesis [34, 35]. However, HIF-1α may also promote maladaptive immune responses by inducing pro-inflammatory cytokines and altering immune cell infiltration [36–38]. Concurrently, HS is frequently accompanied by coagulation dysfunction and fibrinolytic imbalance [39, 40]. Coagulation factors such as F3 (tissue factor), F7, F8, and F10 play critical roles in microvascular thrombosis, endothelial activation, and vascular permeability, serving as potential mediators linking hemorrhage to organ failure [41].

Recently, the gut microbiota has emerged as a key modulator in systemic diseases, including sepsis and critical illness [42, 43]. In HS, intestinal hypoperfusion and barrier disruption facilitate bacterial translocation and endotoxin release, triggering systemic inflammation [44, 45]. Dysbiosis itself may further alter host responses via microbial metabolites, signaling molecules, and hormonal pathways [46, 47]. Although emerging evidence suggests that microbiota may influence the expression of HSPs, apoptotic proteins, HIFs, and coagulation factors, systematic studies addressing this interplay in HS are still lacking [35, 48, 49].

Notably, the molecular regulators investigated here (e.g., HSPB1, HIF1A, APAF1, F7, F10) participate in fundamental stress, apoptosis, hypoxia, and coagulation programs that are conserved across mammalian species, which allows human genetic signals to be mechanistically explored in a rat model. Against this backdrop, we propose a “key protein–microbiota–HS” triadic regulatory hypothesis, postulating that HSPs, apoptotic proteins, HIFs, and coagulation factors may exert their effects on HS progression through specific gut microbial communities [50–52]. Building on our Mendelian randomization results, we hypothesize that heat shock protein beta-1 (HSPB1) and hypoxia-inducible factor 1-alpha (HIF1A) exert their protective associations primarily by stabilizing the intestinal mucosal barrier—via cytoskeletal/tight-junction maintenance and hypoxia adaptation—with systemic anti-inflammatory effects as secondary contributors. Conversely, APAF1-driven epithelial apoptosis and F7/F10-driven microvascular coagulation are expected to alter mucosal oxygen and nutrient landscapes, favoring Proteobacteria expansion and the loss of short-chain-fatty-acid–producing commensals, thereby reshaping gut microbial composition. To test this hypothesis, we employed an integrative strategy combining Mendelian randomization (MR) analysis with in vivo validation. First, genetic instruments for HSPB1, APAF1, HIF1A, and coagulation factors were selected from large-scale genome-wide association studies (GWAS) to infer their causal relationships with HS risk and potential microbial mediation. Second, we established rat HS models with varying degrees of blood loss and performed 16 S rRNA sequencing to assess microbiota composition, diversity, and functional profiles, linking microbial alterations with protein expression pathways [53, 54]. Translationally, we hypothesize that recovery-associated taxa—particularly the rise of Blautia by 72 h and the normalization of Lactobacillus after its 24 h bloom—could serve as stool biomarkers of recovery in HS. We further posit that targeted microbial interventions, such as SCFA supplementation and mechanism-aligned probiotics, may enhance mucosal barrier repair and improve outcomes, which we will prospectively evaluate in follow-up experiments.

This study aims to elucidate the gut microbiota as a potential mediator bridging host stress proteins and systemic responses during HS [55]. By integrating causal inference with experimental validation, we seek to fill a critical knowledge gap in the “host–microbiome–systemic stress” axis and provide a conceptual framework for microbiota-targeted interventions in the management of hemorrhagic shock [56, 57]. Where appropriate, we interpret cross-species consistencies at the pathway level, while explicitly acknowledging species-specific contexts in the Discussion. To prospectively test this protein–microbiota–hemorrhagic shock axis at the protein level, we pre-specified tissue prioritization (colon and ileum mucosa, liver, and lung) and plasma sampling with time-stamped assays at 0, 24, and 72 h.

## Materials and methods

### Population-based cohort data and Mendelian randomization analysis

This study utilized a two-sample Mendelian randomization (MR) framework to systematically investigate the causal relationships between the expression levels of key molecular regulators—including heat shock proteins (HSPB1, HSPA1A, HSPA4), the apoptotic mediator APAF1, the hypoxia-inducible factor HIF1A, and coagulation factors (F3, F7, F8, F10)—and the risk of hemorrhagic shock, suggesting directional effects that require in vivo protein-level validation in the rat model to directly test protein–microbiota coupling. MR leverages genetic variants (single nucleotide polymorphisms, SNPs) as instrumental variables, offering a robust strategy to mitigate confounding and reverse causality inherent in traditional observational research. Genetic instruments for the exposure traits were obtained from large-scale genome-wide association studies (GWAS) of European ancestry. The outcome variable, hemorrhagic shock, was sourced from publicly accessible databases, including the UK Biobank. While the MR instruments and outcomes are derived from human cohorts, the implicated regulators map to rat orthologs with conserved pathway functions, providing a principled basis to prioritize and test mechanisms in vivo. To maximize statistical power and robustness, SNPs showing strong associations with the expression levels of target genes (*p* < 5 × 10⁻⁸) were selected, and linkage disequilibrium (LD) clumping was performed (r² < 0.001; 10,000 kb window) to ensure independence among instruments. Primary causal estimates were derived using the inverse-variance weighted (IVW) method, complemented by MR-Egger regression and the weighted median method for sensitivity and pleiotropy analyses. All analyses were conducted in R using the “TwoSampleMR” package. Statistical significance was defined as a two-sided p-value < 0.05, with Bonferroni correction applied (adjusted threshold: *p* < 0.01). Results are presented as odds ratios (ORs) with 95% confidence intervals and were visually validated using scatter plots, forest plots, funnel plots, and leave-one-out sensitivity plots. All data were obtained from ethically approved public repositories, obviating the need for additional ethical review. For orientation, the conceptual framework that guided variable selection and interpretation is summarized in Supplementary Fig. S4, which depicts the MR-inferred directions between host proteins and hemorrhagic shock (HS) and the hypothesized microbiota mediation.

### Experimental animals and establishment of the hemorrhagic shock model

Eight-week-old male Sprague-Dawley (SD) rats (specific pathogen-free, 200–250 g) were purchased from the Laboratory Animal Center of Nanchang University.

#### Rationale for independent-cohort design and bias control

We prespecified independent cohorts at baseline (A), 24 h (B), and 72 h (C) instead of repeatedly sampling the same rats to: (i) avoid repeated handling/cage changes that acutely perturb fecal microbiota, (ii) prevent confounding from post-hemorrhage care (e.g., analgesia/resuscitation) that differentially affects later time points in a within-subject scheme, and (iii) ensure adequate sample size at 72 h without survivor bias. To mitigate inter-animal variability, rats were randomized, co-housing and cage distribution were balanced, fecal collection followed a standardized protocol by blinded personnel, and “cage” was recorded as a blocking factor for downstream sensitivity analyses. We acknowledge that this independent-cohort design introduces between-animal variability at each time point; therefore, we prespecified dispersion metrics (Bray–Curtis betadisper) and cage-constrained permutations and interpret time effects as group-level differences rather than within-animal changes. All animal procedures were approved by the Animal Ethics Committee of Nanchang University and conducted in strict compliance with the ARRIVE guidelines and the Chinese national regulations for laboratory animal care and use. Anesthesia was induced via intraperitoneal injection of 10% chloral hydrate at a dose of 350–450 mg/kg, adjusted according to body weight. Upon achieving sufficient anesthesia, 4 mL of blood was slowly withdrawn via femoral artery puncture to establish a moderate hemorrhagic shock model. No crystalloid or blood products were given after hemorrhage in this phase to deliberately isolate the ecological impact of hypoperfusion and gut hypoxia on microbiome trajectories. Following blood withdrawal, the rats were placed in a warming chamber for recovery and provided with free access to food and water. Fecal samples were collected at either 24–72 h post-hemorrhage, immediately flash-frozen in liquid nitrogen, and stored at − 80 °C for subsequent microbiome profiling.

#### Study scope and sample allocation

At protocol registration, we pre-specified the primary endpoint as time-resolved microbiome remodeling during HS to build a testable “protein–microbiota–HS” hypothesis aligned with MR inference. Given constraints on animal material, costs, and time windows, available samples were prioritized for 16 S ribosomal RNA gene sequencing and ecological analyses; therefore, tissue and plasma protein assays for heat shock protein beta-1 (HSPB1), hypoxia-inducible factor 1-alpha (HIF1A), apoptotic protease activating factor-1 (APAF1), coagulation factor VII (F7), and coagulation factor X (F10) were not performed in this phase. Furthermore, the study did not assess the long-term complications that may arise after the acute phase (i.e., sepsis from gut translocation). Future studies with extended follow-up periods are required to address these potential complications and their link to microbiome shifts observed in the early stages. Follow-up validation will quantify these proteins at 0, 24, and 72 h in colon and ileum mucosa, liver, lung, and plasma using Western blot for tissue lysates, immunohistochemistry for spatial localization in gut, liver, and lung, and enzyme-linked immunosorbent assay for plasma heat shock protein beta-1 (HSPB1), hypoxia-inducible factor 1-alpha (HIF1A), coagulation factor VII (F7), and coagulation factor X (F10), paired with microbial readouts to directly test protein–microbiota coupling. To address clinical translatability, a preregistered, parallel-group follow-up will directly compare non-resuscitated controls with standard resuscitation arms (isotonic crystalloid and packed red blood cells), targeting restoration of mean arterial pressure and lactate clearance. Prespecified endpoints include attenuated 24-hour declines in alpha diversity, reduced beta-diversity separation, lower relative abundance of Proteobacteria, and earlier recovery of SCFA-associated genera (e.g., Blautia and Romboutsia), accompanied by perfusion/hypoxia readouts (e.g., mucosal pO₂ proxies) at 0, 24, and 72 h. In parallel, we interpret findings within a cross-species framework, emphasizing conserved pathway biology (stress proteins, apoptosis, hypoxia signaling, and coagulation) while avoiding over-extrapolation beyond species boundaries. In exploratory interventional arms, we will administer SCFA supplementation (e.g., butyrate) and a rationally selected probiotic formulation enriched for recovery-associated taxa (e.g., Lactobacillus/Blautia profiles). Primary readouts will include survival, mean arterial pressure, lactate clearance, and pre-specified stool biomarkers (directional changes in Lactobacillus and Blautia trajectories), alongside standard microbiome and perfusion endpoints.

### Fecal DNA extraction and high-throughput sequencing of 16 S rRNA genes

Genomic DNA was extracted from fecal samples using the FastDNA Spin Kit for Feces (MP Biomedicals, USA). DNA purity was evaluated with a NanoDrop 2000 spectrophotometer, and integrity was verified by agarose gel electrophoresis. The V3–V4 hypervariable region of the bacterial 16 S rRNA gene was amplified using universal primers 341 F (5′-CCTACGGGNGGCWGCAG-3′) and 805R (5′-GACTACHVGGGTATCTAATCC-3′). PCR amplification was performed in 25 µL reaction volumes over 30 cycles, and products were purified using the AxyPrep DNA Gel Extraction Kit. Sequencing libraries were constructed and subjected to paired-end 250 bp (PE250) sequencing on the Illumina MiSeq platform. Raw sequencing reads were processed using Trimmomatic and FLASH for quality filtering and merging, with the removal of low-quality reads and chimeric sequences. High-quality reads were denoised using the DADA2 algorithm to generate amplicon sequence variants (ASVs). Taxonomic assignment was conducted using the SILVA v138 reference database, covering six hierarchical levels from phylum to species.

### Microbial community profiling and functional prediction

Alpha diversity metrics were used to evaluate species richness and evenness within individual samples. The Chao1 and ACE indices estimated microbial richness, while the Shannon and Simpson indices measured community evenness.

Beta diversity assessed compositional differences between microbial communities across groups. Sample similarities were calculated using Bray-Curtis distance matrices and visualized via Principal Coordinates Analysis (PCoA) and Non-metric Multidimensional Scaling (NMDS). Statistical significance between groups was determined using PERMANOVA (Permutational Multivariate Analysis of Variance).

For taxonomic composition analysis, relative abundances at the phylum, class, order, family, and genus levels were calculated based on ASV annotations. Stacked bar charts were used to illustrate shifts in dominant taxa. Differentially abundant genera were identified using Linear Discriminant Analysis Effect Size (LEfSe), applying an LDA score threshold > 4.0 and p-value < 0.05 to pinpoint group-specific microbial signatures for downstream interpretation.

To explore functional potential, PICRUSt2 was employed to infer metagenomic functions from ASV sequences. Predicted functions were mapped to level 2 pathways in the KEGG database, enabling the identification of microbial contributions to pathways such as carbohydrate metabolism, lipid biosynthesis, and energy conversion. These findings provide mechanistic insights into the microbiota’s role in modulating host physiological processes.

#### Statistical controls for inter-animal variability

Group differences were tested by PERMANOVA (Bray–Curtis; 9,999 permutations). We additionally assessed within-group dispersion (betadisper) and, when cage records were available, performed permutations constrained within cage strata. Effect sizes (e.g., Hedges’ g for alpha indices) are reported with 95% CIs. Because cohorts were independent, dispersion estimates and effect sizes reflect between-rat heterogeneity and are reported to transparently contextualize the magnitude and uncertainty of time-point contrasts. In planned validation, we will incorporate shotgun metagenomics and metatranscriptomics to directly evaluate pathway enrichment inferred by PICRUSt2, together with targeted metabolomics focusing on short-chain fatty acids, amino acids, and bile acids to examine metabolite outputs; all validations will be aligned to the 0-hour, 24-hour, and 72-hour time points and will use colon mucosa, ileum mucosa, feces, and plasma to ensure consistency with the present study design.

## Results

### Summary of sequencing output

A total of 24 intestinal samples from rats were subjected to 16 S rRNA high-throughput sequencing, representing three experimental groups: pre-hemorrhage (Group A), 24 h post-hemorrhage (Group B), and 72 h post-hemorrhage (Group C). The raw read counts per sample ranged from 71,000 to 80,000. After quality filtering, both clean reads and denoised reads remained consistently around 72,000, indicating sufficient sequencing depth. Merged reads averaged approximately 70,000 per sample, and the final number of high-quality non-chimeric reads ranged from 64,000 to 67,000, accounting for more than 80% of the initial raw reads (Table S5). These metrics indicate that the sequencing data were of high quality and suitable for robust analysis of microbial community structure and functional potential.

**Fig. 1:**
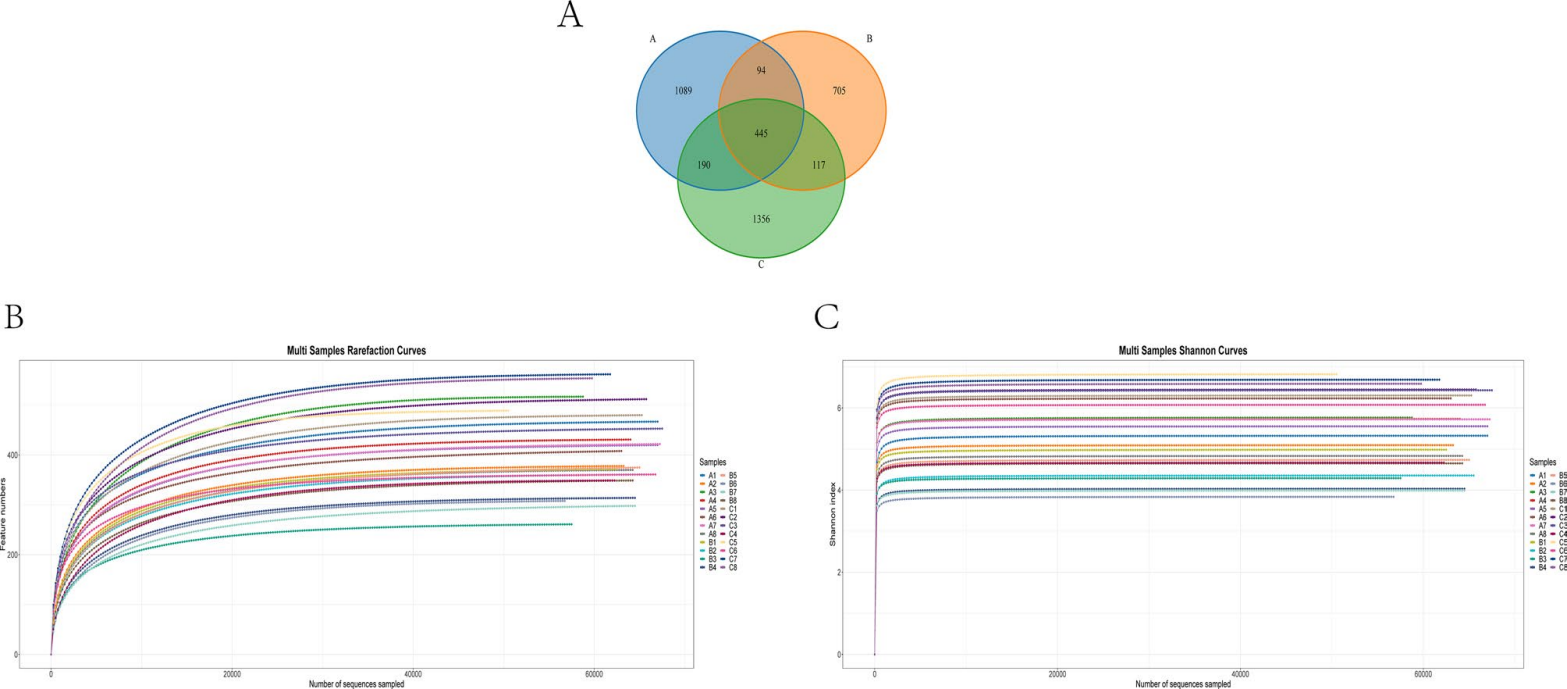
Microbiota sequencing in bloodletting mice

As shown in Fig. 1A, Venn diagrams of the three groups reveal both shared and unique features of the gut microbiota. A total of 445 operational taxonomic units (OTUs) were common across all groups, constituting the core microbiota. This pattern suggests that over time following hemorrhage, microbial community overlap declines while compositional specificity increases. This trend is further supported by the petal diagram in Figure S3: the number of core OTUs decreased from 80 in Group A to 63 in Group B and dropped to 50 in Group C. Additionally, substantial variation in unique OTUs was observed among individual samples—sample C8 alone exhibited 288 unique OTUs—indicating a marked rise in microbiota individualization 72 h after hemorrhage (Figure S3).

Rarefaction curve analysis (Fig. 1B) demonstrated that sequencing depth was adequate, as all samples reached a plateau beyond 40,000 reads, capturing the vast majority of community members. Shannon index rarefaction curves (Fig. 1C) highlighted dynamic shifts in microbial diversity: Group A displayed the highest diversity, suggesting optimal richness and evenness; Group B showed the lowest, reflecting a sharp diversity decline at 24 h post-hemorrhage; while Group C exhibited partial recovery, the large inter-individual variation implied persistent instability—likely representing a transitional phase of microbial reconstruction or stress adaptation.

Collectively, the sequencing dataset met high standards in depth, accuracy, and coverage. With increasing hemorrhage severity and time, the gut microbiota underwent a progressive loss of diversity and ecological balance, shifting from a stable, highly shared community structure toward one characterized by heightened specificity and dysbiosis. The corresponding functional modules (e.g., increased translation and amino-acid metabolism at 72 h) are summarized in Supplementary Fig. S4 as conceptual, hypothesis-generating links to HS.

### Microbial diversity analysis highlights pronounced shifts and remodeling of the gut microbiota

**Fig. 2:**
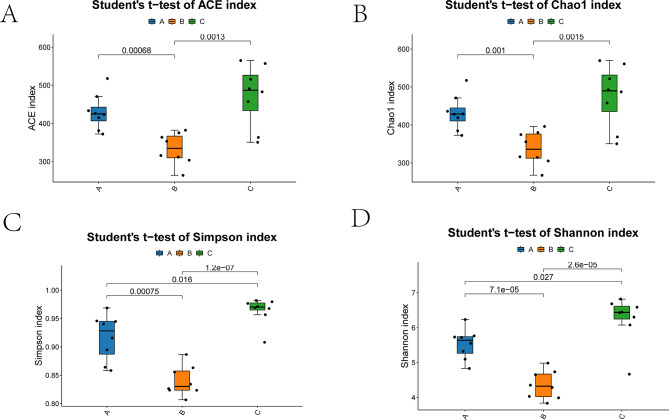
Gut_microbiota diversity in graded bloodletting mice

Alpha and beta diversity analyses were performed to systematically assess the impact of hemorrhagic shock on gut microbial diversity and community architecture. At this discovery stage, we did not quantify protein expression in rat tissues; accordingly, the Results focus on microbiome ecology integrated with MR-inferred directions, with direct protein–microbiota coupling reserved for planned validation. Because an independent-cohort design was used, we report dispersion metrics and cage-stratified sensitivity analyses to contextualize inter-animal variability at each time point, and we note that between-animal heterogeneity may widen confidence intervals and attenuate some group contrasts. As no resuscitation was administered, these trajectories likely reflect unmitigated hypoperfusion and gut hypoxia; based on this premise we anticipate that standard resuscitation will lessen the 24-hour drop in alpha diversity, narrow between-group separation in beta diversity, curb expansion of stress-responsive Proteobacteria, and hasten the rebound of SCFA-associated commensals by 72 h—effects that will be evaluated in the preregistered resuscitation arm. For alpha diversity, we evaluated ACE (Fig. 2A) and Chao1 (Fig. 2B) indices reflecting species richness, and Simpson (Fig. 2C) and Shannon (Fig. 2D) indices reflecting microbial diversity and evenness (Table S6). Statistical significance is denoted as *p < 0.05, **p < 0.01, ***p < 0.001. At 24 h post-hemorrhage (Group B), a pronounced decline in microbial richness and evenness was observed. Both ACE and Chao1 indices were significantly reduced in Group B compared to Group A (*p* = 0.00068 and 0.001, respectively), indicating a sharp drop in species richness. The Simpson index also decreased (Fig. 2C), suggesting dominance by a limited number of taxa. The Shannon index, which integrates richness and evenness, was significantly lower in Group B (*p* = 7.1 × 10⁻⁵), with the lowest value recorded in sample B6 (3.83), well below that of Groups A and C (Fig. 2D, Table S6). By 72 h (Group C), all indices showed signs of recovery, with some samples exceeding baseline levels, indicating potential for microbial self-restoration or compensatory adaptation.

**Fig. 3:**
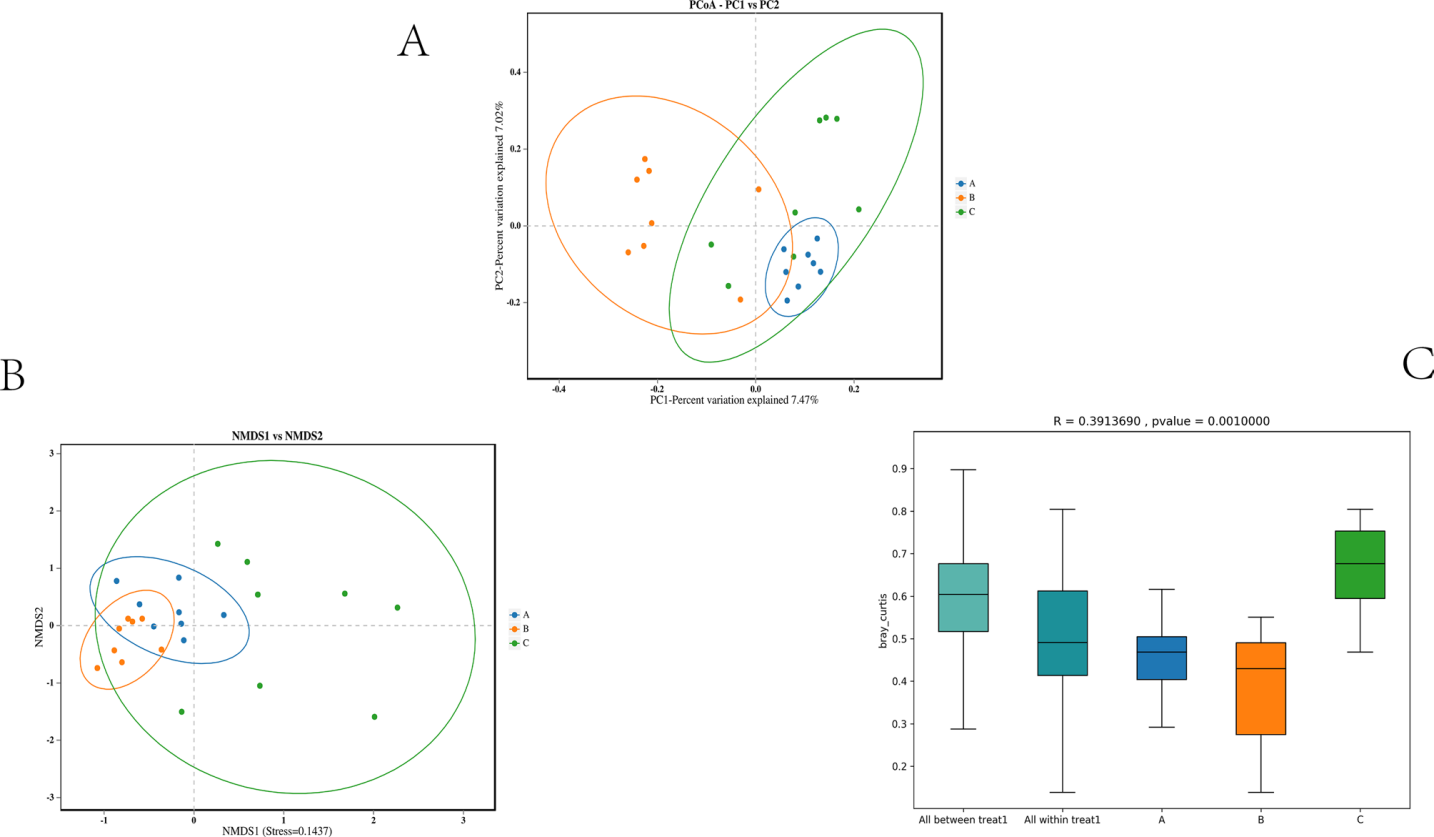
Gut microbiota community in graded bloodletting mice

Beta diversity analysis was performed using Bray–Curtis dissimilarity, visualized by Principal Coordinates Analysis (PCoA; Fig. 3A), Non-metric Multidimensional Scaling (NMDS; Fig. 3B), and PERMANOVA significance testing (Fig. 3C). These analyses highlight group-specific clustering, with clear separation among baseline, 24 h, and 72 h samples. The first two principal coordinates (PC1 and PC2) accounted for 14.49% of the total variance, with clear separation among the three groups in ordination space (Fig. 3A). Samples from Group B were tightly clustered, reflecting homogenization, whereas Group C samples were more dispersed, indicative of greater inter-individual variability and potentially asynchronous recovery (Fig. 3B). Group-level Bray-Curtis distances were smallest in Group B and largest in Group C, reinforcing these observations (Fig. 3B, Table S6). PERMANOVA confirmed significant compositional differences among groups (*R* = 0.3914, *p* = 0.001; Fig. 3C). Given the independent-cohort design, the larger dispersion by 72 h indicates increased between-rat heterogeneity, which could obscure subtle within-animal recovery trajectories that a longitudinal design would be powered to detect.

Collectively, alpha and beta diversity analyses converge to demonstrate that hemorrhagic shock causes acute disruption of gut microbial diversity and ecological stability. The 24-hour period represents a phase of intense perturbation, followed by partial recovery by 72 h, suggesting that the gut microbiota is sensitive to hemorrhagic stress yet possesses a time-dependent potential for ecological resilience and reconstruction.

### Shifts in gut microbial community composition

Hemorrhagic shock induced significant alterations in the gut microbial community structure of rats, with time-dependent shifts observed at both the phylum and genus levels.

**Fig. 4:**
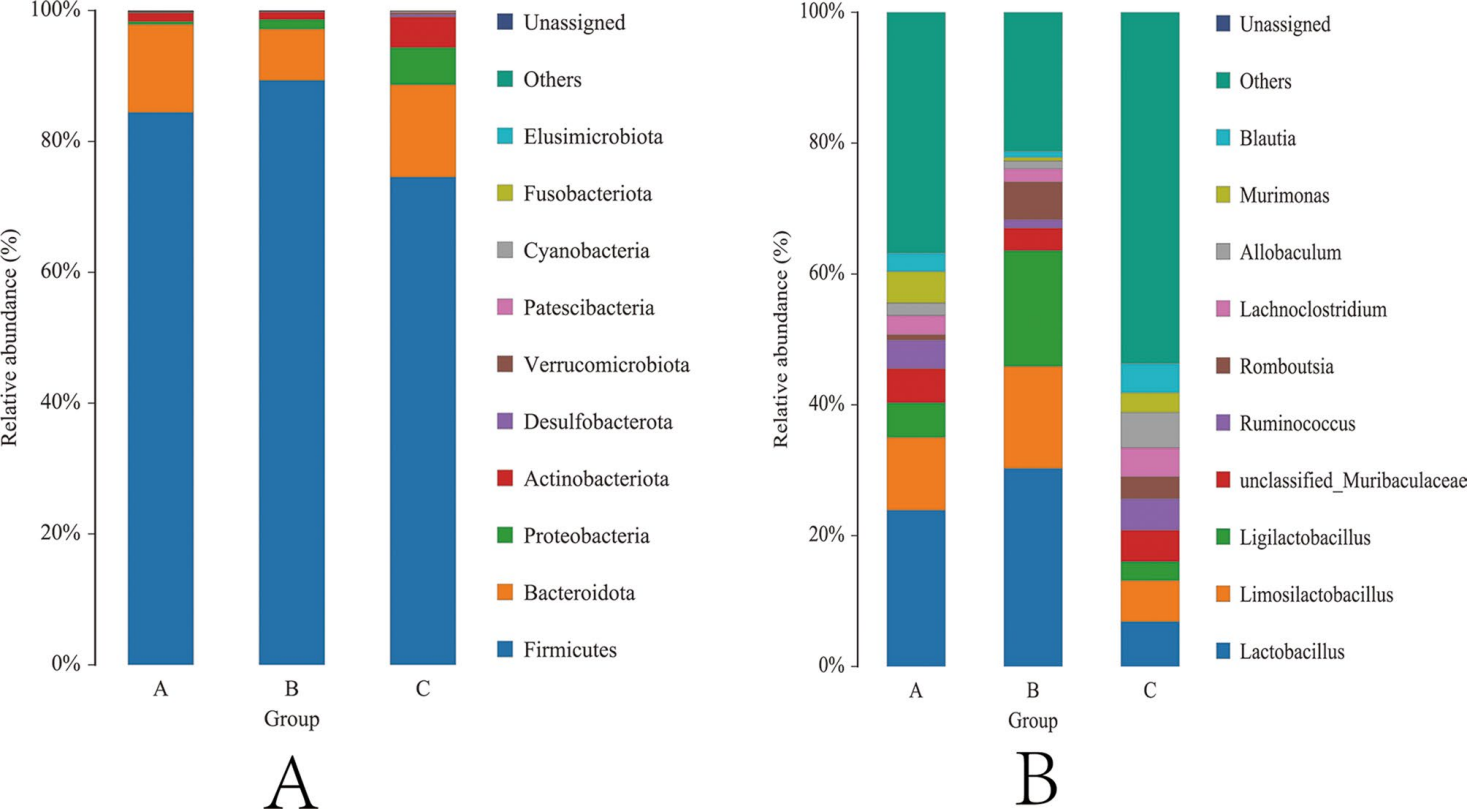
Gut microbiota taxonomy in graded bloodletting mice

**Fig. 5:**
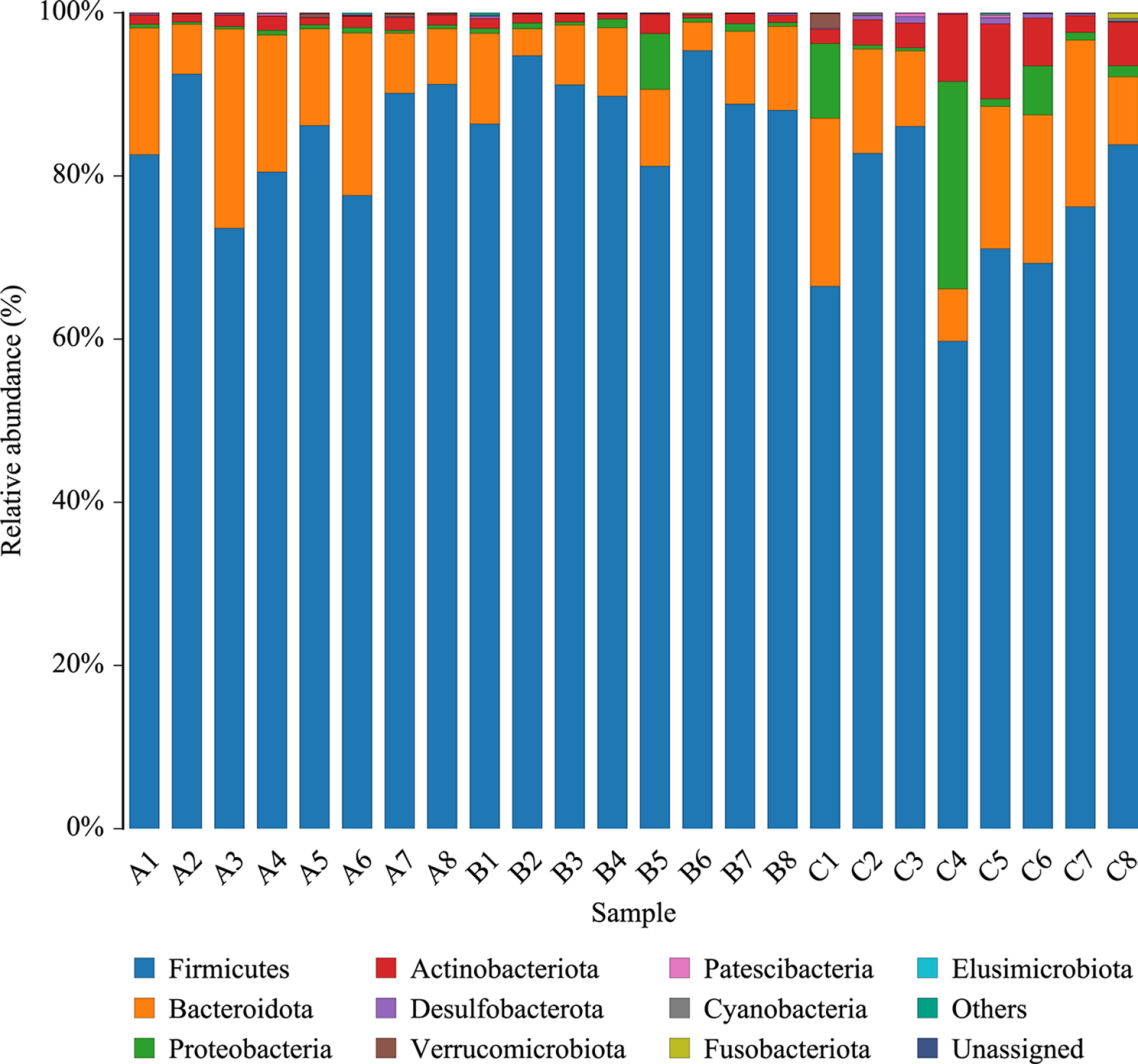
Phylum diff in graded bloodletting mice

At the phylum level, relative abundances of the top 10 bacterial phyla are shown (Fig. 4A), while genus-level relative abundances are displayed (Fig. 5). Stacked bar plots illustrate the compositional shifts among baseline, 24 h, and 72 h groups, with key phyla and genera labeled in the figures, Firmicutes remained dominant across all groups but declined substantially in Group C at 72 h post-hemorrhage (74.55%), compared to 84.43% in Group A and 89.33% in Group B (Table 1), indicating a gradual erosion of its ecological dominance. Meanwhile, Bacteroidota showed a compensatory increase in Group C (14.14%) versus Group A (13.44%) and Group B (7.82%). Strikingly, Proteobacteria (A: 0.28%, B: 1.47%, C: 5.65%) and Actinobacteriota (A: 1.03%, C: 4.60%) were significantly elevated in Group C, with some individual samples (e.g., C1, C4) exceeding 10% in combined abundance (Fig. 5). These findings may reflect expansion of potentially pathogenic taxa during the late stages of stress; however, barrier dysfunction or secondary infections were not directly assessed, and this interpretation is hypothesis-generating.

**Table 1:** Relative abundance of the top 10 microbial phyla in mice exposed to graded volumes of bloodletting

Top 10 Phylum	A	B	C
Firmicutes	0.84433	0.893273	0.745475
Bacteroidota	0.134429	0.078274	0.141373
Proteobacteria	0.004501	0.014742	0.056547
Actinobacteriota	0.012891	0.01133	0.046907
Desulfobacterota	0.000491	0.001049	0.003695
Verrucomicrobiota	0.001436	2.79E-05	0.002518
Patescibacteria	0.00092	0.000277	0.001249
Cyanobacteria	0.000542	0.000116	0.001107
Fusobacteriota	0	0.000194	0.000781
Elusimicrobiota	0.000382	0.000313	0.000234
Others	7.76E-05	0.000403	0.000102
Unassigned	0	0	1.20E-05

Note: Top 10 Genera — the most abundant bacterial genera across samples; Others — the combined relative abundance of all genera outside the top 10; Unassigned — sequences that could not be classified into any known genus; E–05 — scientific notation denoting 10⁻⁵; A — baseline (prior to bloodletting); B — 24 h after bloodletting; C — 72 h after bloodletting

At the genus level, further functional restructuring was evident (Fig. 4B; Table 2). Group A was enriched with commensal genera commonly linked to symbiosis, immunomodulation, and SCFA production, including Lactobacillus (23.91%), Ruminococcus (4.33%), Allobaculum (3.62%), Murimonas (4.76%), and Lachnoclostridium (2.91%). In Group B, Lactobacillus exhibited a sharp rise to 30.29%, consistent with a stress-associated bloom, while other beneficial genera such as Allobaculum (1.02%) and Murimonas (0.52%) declined. By Group C, Lactobacillus abundance dropped significantly to 6.86%, whereas Romboutsia (3.36%), Blautia (4.43%), and Murimonas (2.98%) increased, suggesting an ongoing functional reorganization of the microbial community. Nonetheless, most of the dominant genera observed in Group A did not return to baseline levels. Notably, the “Others” category—comprising low-abundance and unclassified taxa—rose sharply in Group C to 53.72% (Table 2), indicating opportunistic expansion and heightened inter-individual variability, along with increased microbial complexity.

**Table 2:** Relative abundance of the top 10 bacterial genera in mice exposed to graded volumes of bloodletting

Top 10 Genera	A	B	C
Lactobacillus	0.239087	0.302862	0.068558
Limosilactobacillus	0.111071	0.156046	0.062817
Ligilactobacillus	0.052883	0.176869	0.029043
unclassified_Muribaculaceae	0.052452	0.034573	0.047904
Ruminococcus	0.043331	0.012639	0.048326
Romboutsia	0.008485	0.057756	0.033588
Lachnoclostridium	0.02912	0.01973	0.043987
Allobaculum	0.019491	0.012444	0.054359
Murimonas	0.047638	0.005175	0.029807
Blautia	0.028072	0.008765	0.044383
Others	0.368369	0.213142	0.537217
Unassigned	0	0	1.20E-05

Note: Top 10 Genera — the most dominant bacterial genera identified across samples; Others — combined relative abundance of all genera not ranked in the top 10; Unassigned — sequences unclassified at the genus level; E–05 — scientific notation indicating 10⁻⁵; A — baseline (prior to bloodletting); B — 24 h after bloodletting; C — 72 h after bloodletting

### LEfSe- and PICRUSt2-based profiling of differentially abundant genera and their predicted metabolic functional shifts

**Fig. 6:**
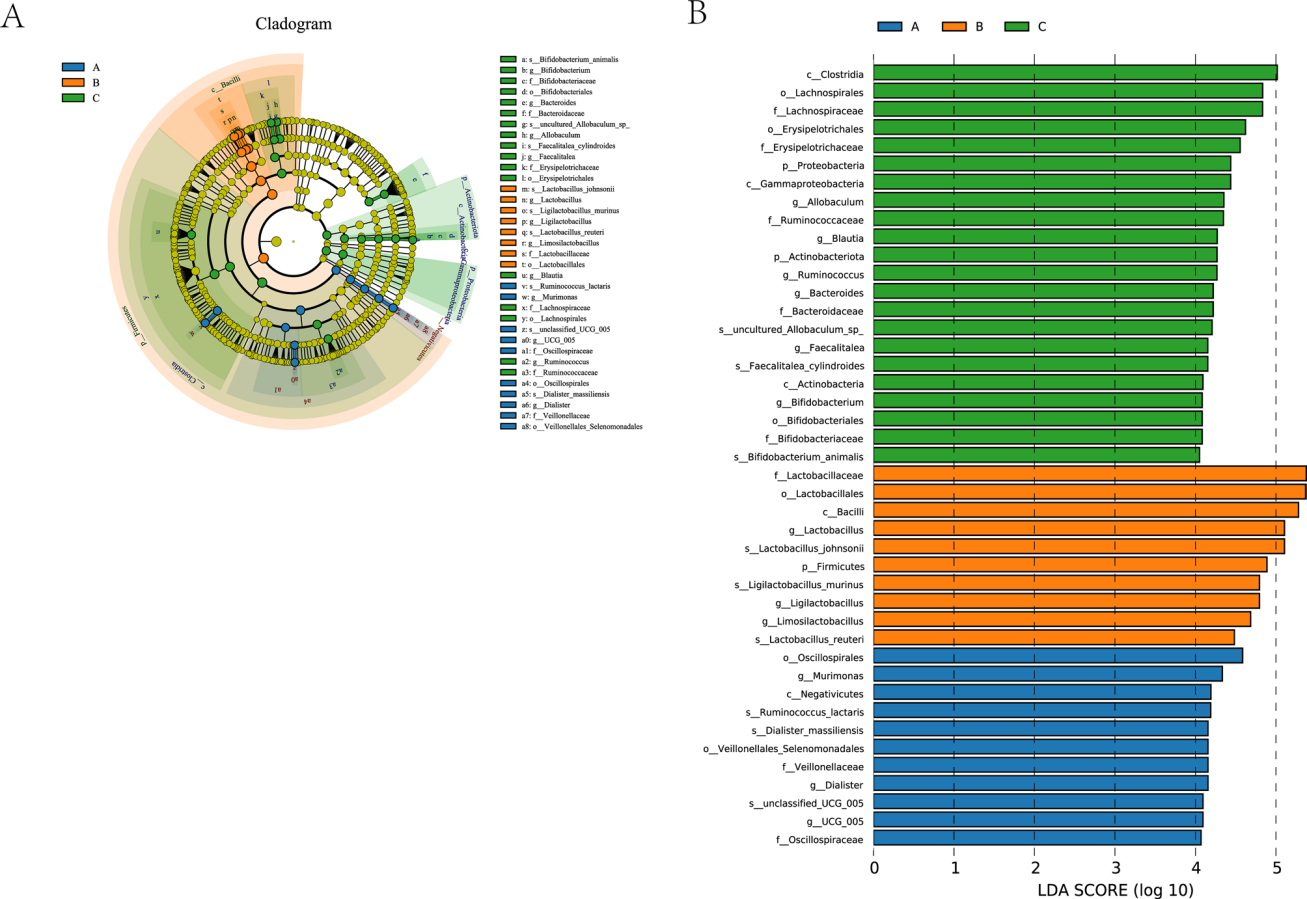
LEfSe analysis in bloodletting mice

Integrated LEfSe analysis identified differentially abundant taxa (Fig. 6), with cladogram visualization of phylogenetic distribution (panel A) and LDA scores > 4.0 highlighting discriminative taxa for each group (panel B). Colors indicate the group in which each taxon was enriched. Differentially abundant genera were primarily enriched within the phyla Firmicutes and Actinobacteriota, with transient proliferation of Proteobacteria, collectively reflecting a dynamic microbial trajectory from homeostasis to acute stress and onward to ecological reconstruction (Fig. 6).

Before hemorrhage, the gut microbiome was dominated by anaerobic symbionts such as Veillonellaceae, Dialister, Murimonas, and Ruminococcus_lactaris, taxa involved in short-chain fatty acid (SCFA) production and immune homeostasis. At 24 h post-hemorrhage, rapid expansion of Lactobacillus, Ligilactobacillus, and Limosilactobacillus was observed. While these taxa have been implicated in metabolic and barrier functions in prior studies, their roles were not directly tested here and are interpreted descriptively. By 72 h, the microbiota exhibited increased diversity, with phylogenetically clustered enrichment of genera such as Blautia, Romboutsia, Ruminococcus, and Erysipelotrichaceae, indicative of a transition toward community-level reconstruction. However, a notable rise in Proteobacteria, particularly Gammaproteobacteria, at this stage implied that microbiota stability had yet to be fully reestablished and potential immune risk remained.

**Fig. 7:**
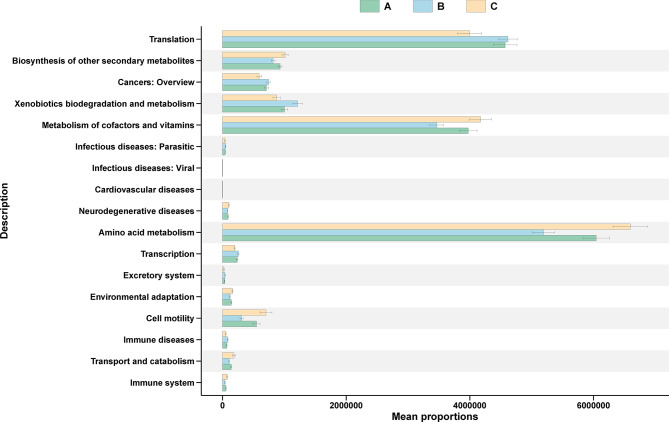
KEGG L2 pathways in graded bloodletting mice

**Fig. 8:**
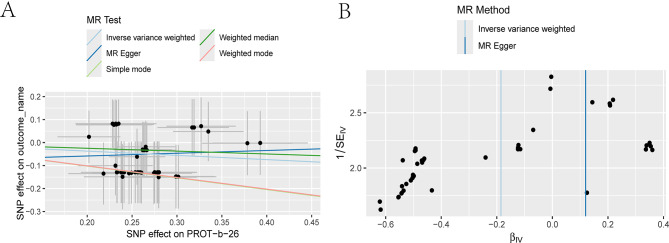
HSPB1 scatterfunnel plots in hemorrhagic shock

Functional predictions inferred by PICRUSt2 are presented in Fig. 7, with KEGG level 2 pathways shown (panel A) and stage-specific enrichment of translation and amino acid metabolism pathways at 72 h highlighted (panel B). These results are predictive and hypothesis-generating, requiring further validation. Pathways related to Translation and Amino Acid Metabolism were among the most abundant across all groups and peaked at 72 h (Group C), indicating higher PICRUSt2-predicted capacities for protein synthesis and amino acid turnover; these predictions are hypothesis-generating and require further metabolomic or protein-level validation. Collectively, these findings indicate that hemorrhagic stress induces phased and coordinated alterations in gut microbiota composition and function, tracing a continuum from acute stress response to early-stage ecological and metabolic reconstruction. Accordingly, we will validate the predicted enrichment of pathways related to translation and amino acid metabolism, and the predicted increases in short-chain fatty acid biosynthesis, by conducting shotgun metagenomics and metatranscriptomics at matched time points and by measuring short-chain fatty acids, amino acids, and bile acids with targeted metabolomics in colon mucosa, ileum mucosa, feces, and plasma see Fig. 8.

### MR-inferred genetic associations between key regulatory proteins and hemorrhagic shock

To explore whether key stress-related proteins exert genetic influence on the risk of hemorrhagic shock, Mendelian randomization (MR) analyses were performed focusing on APAF1, a central apoptotic regulator, and HIF1A, a hypoxia-inducible transcription factor. MR analyses were conducted using standard frameworks to estimate genetic associations, with supporting visualizations via scatter and funnel plots (Tables 5–7; Figures S1–S2).

As shown in Table 3, genetically proxied APAF1 was associated with a higher risk of hemorrhagic shock (b = 0.212, *p* = 0.004, OR = 1.236, 95% CI: 1.069–1.428), whereas HIF1A was associated with a lower risk (b = − 0.122, *p* = 0.001, OR = 0.684, 95% CI: 0.538–0.869). These MR-inferred associations are consistent with their reported biological roles, although protein-level mechanisms were not directly tested in the present study.

**Table 3:** Mendelian randomization analysis of significant genes associated with hemorrhagic shock under different exposure conditions

gene	outcome	method	nsnp	b	se	pval	lo_ci	up_ci	or	or_lci95	or_uci95
HSPB1	Hemorrhagic shock	Inverse variance weighted	44	-0.1851	0.071272	0.009403	-0.32479	-0.0454	0.831024	0.72268	0.955611
APAFI	Hemorrhagic shock	Inverse variance weighted	46	0.212019	0.073769	0.004052	0.067433	0.356606	1.236172	1.069759	1.428472
HIFIA	Hemorrhagic shock	Inverse variance weighted	54	-0.37904	0.122326	0.001944	-0.6188	-0.13928	0.684516	0.538589	0.869981
F8	Hemorrhagic shock	Inverse variance weighted	135	-0.29576	0.038506	1.58E-14	-0.37123	-0.22029	0.743966	0.689884	0.802287
F10	Hemorrhagic shock	Inverse variance weighted	159	0.1656	0.038062	1.36E-05	0.090998	0.240202	1.180101	1.095267	1.271506
F7	Hemorrhagic shock	Inverse variance weighted	481	0.073083	0.029792	0.014164	0.01469	0.131476	1.075819	1.014798	1.14051

Note: nsnp — number of instrumental SNPs used in the analysis; b — regression coefficient, indicating the effect size and direction of the exposure on the outcome; se — standard error of the coefficient, reflecting estimate uncertainty; pval — p-value evaluating the statistical significance of the association; lo_ci / up_ci — lower and upper limits of the 95% confidence interval for b; or — odds ratio, derived from b, representing the relative risk associated with the exposure; or_lci95 / or_uci95 — lower and upper limits of the 95% confidence interval for the odds ratio

Robustness assessments showed no evidence of heterogeneity based on Cochran’s Q tests in both IVW and MR Egger models (APAF1: Q_pval = 1; HIF1A: Q_pval = 0.8419; Table 4), indicating consistent instrument performance. Additionally, MR Egger intercepts were not statistically significant (APAF1: intercept = 0.036, *p* = 0.5639; HIF1A: intercept = − 0.0845, *p* = 0.2049; Table 5), suggesting minimal horizontal pleiotropy and reducing concern for unbalanced pleiotropy and supporting the robustness of the MR-inferred associations; however, causal inference awaits protein-level validation.

**Table 4:** Heterogeneity analysis of exposure variables associated with hemorrhagic shock

gene	outcome	method	Q	Q_df	Q_pval
HSPB1	Hemorrhagic shock	MR Egger	20.86737	42	0.997405
HSPB1	Hemorrhagic shock	Inverse variance weighted	21.24432	43	0.99782
APAFI	Hemorrhagic shock	MR Egger	4.207802	44	1
APAFI	Hemorrhagic shock	Inverse variance weighted	4.545796	45	1
HIFIA	Hemorrhagic shock	MR Egger	40.02458	52	0.887266
HIFIA	Hemorrhagic shock	Inverse variance weighted	42.74778	53	0.84194
F8	Hemorrhagic shock	MR Egger	29.44322	133	1
F8	Hemorrhagic shock	Inverse variance weighted	29.44358	134	1
F10	Hemorrhagic shock	MR Egger	73.14798	157	1
F10	Hemorrhagic shock	Inverse variance weighted	79.08405	158	1
F7	Hemorrhagic shock	MR Egger	498.6952	479	0.25808
F7	Hemorrhagic shock	Inverse variance weighted	498.8278	480	0.267186

Note: gene – gene serving as the source of instrumental variables or representing the exposure trait in the MR analysis; outcome – the outcome of interest; in this study, hemorrhagic shock; method – analytical approach, typically Mendelian Randomization (MR); Q – Cochran’s Q statistic, used to evaluate heterogeneity among SNP effect estimates; higher values indicate increased heterogeneity; Q_df – degrees of freedom for the Q test, calculated as the number of SNPs minus one; Q_pval – p-value for the Q statistic; *p* < 0.05 denotes statistically significant heterogeneity among SNP effects

**Table 5:** Pleiotropy analysis of exposure variables associated with hemorrhagic shock

gene	outcome	egger_intercept	se	pval
HSPB1	Hemorrhagic shock	-0.08223	0.133935	0.542548
APAFI	Hemorrhagic shock	0.036312	0.062458	0.563956
HIFIA	Hemorrhagic shock	-0.08455	0.051236	0.104928
F8	Hemorrhagic shock	-0.00073	0.03849	0.984902
F10	Hemorrhagic shock	0.129401	0.053111	0.015952
F7	Hemorrhagic shock	0.006668	0.018686	0.721377

Note: egger_intercept – intercept from the MR-Egger regression, used to detect horizontal pleiotropy; a significant deviation from zero indicates potential directional pleiotropy. se – standard error of the intercept estimate, reflecting uncertainty. pval – p-value testing whether the intercept significantly differs from zero

In visual analyses, scatter plots (Figures S1-A and S2-A) demonstrated close alignment of SNP-specific effects with IVW and MR Egger regression lines, supporting the assumption of linearity. Funnel plots (Figures S1-B and S2-B) showed symmetrical distribution without signs of systematic bias, further supporting the robustness of the MR results; no external replication or human validation was performed in this study. An integrated schematic summarizing the MR-inferred directions (protective: HSPB1, HIF1A; risk: APAF1, F7, F10) and the hypothesized microbiota mediation is provided in Supplementary Fig. S4.

### Discussion

Based on the time-resolved patterns observed here, we expect microbial functions most critical for recovery after hemorrhagic shock to include: short-chain fatty acid production, especially acetate, propionate, and butyrate, which supports epithelial barrier repair and hypoxia adaptation; amino acid and nitrogen metabolism, including urea cycle components and branched-chain amino acid catabolism, which buffers nitrogen flux and fuels mucosal regeneration; bile acid transformation, which modulates host–microbe signaling and inflammatory tone; and oxidative-stress mitigation, which may influence both tissue injury and coagulation. These working expectations are aligned with our sampling at 0 h, 24 h, and 72 h and will be examined with shotgun metagenomics, metatranscriptomics, and targeted metabolomics using colon mucosa, ileum mucosa, feces, and plasma. Mechanistic implications (hypothesis-generating). Consistent with our Mendelian randomization findings, we hypothesize that HSPB1 promotes cytoskeletal resilience and tight-junction integrity and that HIF1A sustains epithelial hypoxia adaptation and metabolic reprogramming; together these actions primarily protect the mucosal barrier, while secondarily dampening systemic inflammation. In contrast, APAF1-mediated epithelial apoptosis compromises barrier function and releases danger-associated signals, and coagulation factor activity (F7/F10) promotes microvascular thrombosis and mucosal hypoperfusion, jointly shifting luminal redox and nutrient conditions to favor facultative anaerobes (e.g., Proteobacteria) and deplete short-chain-fatty-acid–producing taxa (e.g., Blautia/Romboutsia)—an ecological trajectory aligned with our time-resolved community changes. Accordingly, we predefine directional thresholds for candidate recovery biomarkers at the genus level—Blautia increase by 72 h and normalization of Lactobacillus after the 24 h bloom—for prospective validation. We also prespecify targeted interventions (SCFA supplementation and rationally selected probiotics) to test whether augmenting these functions improves physiologic and microbiome endpoints.

This study is the first to integrate multi-omics Mendelian randomization (MR) analysis with gut microbiota sequencing in a hemorrhagic shock (HS) rat model to systematically investigate the genetic causal links between key stress-responsive proteins—including HSPB1, APAF1, HIF1A, F7, F8, and F10—and the risk of HS [58–60]. To aid interpretation, a conceptual schematic (Supplementary Fig. S4) depicts the proposed protein–microbiota–HS regulatory axis derived from the current data. Our findings revealed significant protective associations for HSPB1 and HIF1A (OR = 0.831 and 0.684, respectively; *p* < 0.01), while APAF1, F7, and F10 were identified as positive risk factors (OR = 1.236, 1.076, and 1.179; *p* < 0.05), implicating distinct biological mechanisms such as anti-apoptotic defense, hypoxia adaptation, and coagulation regulation in the pathogenesis of HS.

#### Cross-species conservation and translational framing

There is an inherent gap between human genetic inference and rat experimentation. Nevertheless, the regulators and pathways highlighted here—heat shock responses (e.g., HSPB1), hypoxia/HIF signaling (HIF1A), apoptosis (APAF1), and the extrinsic coagulation cascade (F7/F10)—constitute fundamental mammalian programs with conserved molecular architectures and functions. We therefore interpret convergence across datasets at the pathway level as supportive of translational relevance, while explicitly reserving protein-level validation and human cohort testing to confirm effect sizes, directions, and clinical utility. Building on these genetic signals, we next map mechanism-aligned microbiome targets and timing windows that are directly testable in translational studies. Integrating these genetic associations with our rat microbiome trajectories, we prioritize mucosal barrier protection as the dominant protective mechanism for HSPB1 and HIF1A, with systemic anti-inflammatory effects considered secondary.

These causal inferences demonstrated robust statistical support: no evidence of heterogeneity was observed via Cochran’s Q tests (*p* >0.25), and MR Egger intercepts showed no signs of directional pleiotropy (*p* >0.2). Scatter plots exhibited clear linear fits, and funnel plots were symmetrically distributed (Figures S1–S2; Tables 6, and 7), reinforcing the stability and reliability of the MR results [61–63].These findings are further supported by prior studies, including Lee et al.’s demonstration of HSPB1’s anti-inflammatory and mucosal protective roles (Front Immunol, 2020), Wang et al.’s characterization of HIF1A in hypoxia-driven metabolic regulation (PNAS, 1995), and Morishita et al.’s elucidation of APAF1’s role in apoptosis (Sci Rep, 2018), collectively providing strong genetic validation for the observed protein–HS associations [64–66].

**Table 6:** Heterogeneity analysis of various exposure factors and hemorrhagic shock

**gene**	**outcome**	**method**	**Q**	**Q_df**	**Q_pval**
HSPB1	Hemorrhagic shock	MR Egger	20.86737	42	0.997405
HSPB1	Hemorrhagic shock	Inverse variance weighted	21.24432	43	0.99782
APAFI	Hemorrhagic shock	MR Egger	4.207802	44	1
APAFI	Hemorrhagic shock	Inverse variance weighted	4.545796	45	1
HIFIA	Hemorrhagic shock	MR Egger	40.02458	52	0.887266
HIFIA	Hemorrhagic shock	Inverse variance weighted	42.74778	53	0.84194
F8	Hemorrhagic shock	MR Egger	29.44322	133	1
F8	Hemorrhagic shock	Inverse variance weighted	29.44358	134	1
F10	Hemorrhagic shock	MR Egger	73.14798	157	1
F10	Hemorrhagic shock	Inverse variance weighted	79.08405	158	1
F7	Hemorrhagic shock	MR Egger	498.6952	479	0.25808
F7	Hemorrhagic shock	Inverse variance weighted	498.8278	480	0.267186

Note: gene: The gene used as the source of instrumental variables in the analysis or the target exposure gene; outcome: The outcome variable, in this case, hemorrhagic shock; method: The analytical method used, typically MR (Mendelian randomization); Q: Cochran's Q statistic, used to assess whether heterogeneity exists between the effects of different SNPs. A larger value indicates a higher likelihood of heterogeneity; Q_df: The degrees of freedom for the Q test, usually calculated as the number of SNPs used minus 1; Q_pval: The p-value of the Q test. If *p* < 0.05, it indicates statistically significant heterogeneity among the effects of the SNPs

Crucially, this study provided experimental evidence for the microbiome-level response to genetically inferred protein signals by integrating high-throughput 16 S rRNA sequencing with PICRUSt2-based functional prediction [58]. At 24 h post-hemorrhage (Group B), a dramatic decline in microbial alpha diversity was observed (Chao1 dropped from 433.5 to 335.5; Shannon index reached a nadir of 3.83). Although Firmicutes remained dominant (89.33%), Lactobacillus underwent rapid expansion to 30.29%, reflecting a collapse of the symbiotic niche and a prototypical bloom of “acute stress-expansion genera.” By 72 h (Group C), community diversity began to recover, with Lactobacillus decreasing to 6.86% and metabolically functional genera such as Blautia, Romboutsia, and Murimonas gradually re-emerging (Figs. 4 and 5; Tables 1 and 2). These dynamic trajectories nominate the decrease of Lactobacillus from its 24 h bloom and the rise of Blautia by 72 h as candidate stool biomarkers of recovery to be prospectively tested. These stage-specific shifts are mapped onto the proposed protein–microbiota–HS axis in Supplementary Fig. S4 (baseline → 24 h → 72 h).

**Table 7:** Pleiotropy analysis of various exposure factors and hemorrhagic shock

gene	outcome	egger_intercept	se	pval
HSPB1	Hemorrhagic shock	-0.08223	0.133935	0.542548
APAFI	Hemorrhagic shock	0.036312	0.062458	0.563956
HIFIA	Hemorrhagic shock	-0.08455	0.051236	0.104928
F8	Hemorrhagic shock	-0.00073	0.03849	0.984902
F10	Hemorrhagic shock	0.129401	0.053111	0.015952
F7	Hemorrhagic shock	0.006668	0.018686	0.721377

Note: egger_intercept: The MR-Egger intercept term, used to detect horizontal pleiotropy. If the intercept significantly deviates from 0, it indicates the presence of systematic pleiotropy; se: Standard Error, representing the uncertainty of the intercept estimate; pval: The p-value, indicating whether the MR-Egger intercept significantly deviates from 0

LEfSe analysis further revealed that this microbial succession aligned with the depletion of symbionts associated with HSP and HIF signaling (e.g., Veillonellaceae, Ruminococcus) and concurrent activation of APAF1-linked pro-inflammatory pathways (Fig. 6), forming a coordinated network of apoptosis, inflammation, microbial turnover, and functional compensation. These components are integrated in Supplementary Fig. S4 to provide a visual framework linking host proteins, microbial composition, and predicted functions; dashed arrows denote hypothesized mediation requiring protein-level and human validation. Functional predictions from PICRUSt2 showed significant upregulation of translation and amino acid metabolism pathways in Group C (Fig. 7), potentially reflecting HIF1A-guided recovery of protein and nitrogen metabolic capacities. Concurrent enhancement of pathways related to secondary metabolite biosynthesis, environmental adaptation, and xenobiotic degradation suggests that by 72 h post-hemorrhage, the gut microbiome had entered a critical window of functional reconstruction [67].

Our integrative MR and microbiome findings generate several hypothesis-generating avenues for translation to clinical practice. Diagnostics. The MR-inferred associations—APAF1 and coagulation factors (F7/F10) with higher HS risk and HSPB1/HIF1A with lower risk—nominate a candidate blood-based panel (APAF1, HSPB1, HIF1A, F7, F10) complemented by markers of coagulation activation (e.g., TAT complex, D-dimer) and stool-based microbiome metrics (24- to 72-hour changes in alpha diversity and taxa such as *Lactobacillus* and *Blautia*). We will prospectively evaluate these taxa as predefined biomarkers of recovery with directional expectations (Lactobacillus normalization; Blautia increase by 72 h) and report discrimination metrics against clinical endpoints. In parallel, targeted microbial interventions are incorporated into our staged validation plan (see Methods) to assess SCFA supplementation and probiotic strategies for outcome improvement.Such a multimarker approach could augment existing vitals and laboratory indices for early triage and risk stratification in HS. Prediction and monitoring. A multimodal risk score integrating genetic proxies, protein measurements, and microbiome features could be prospectively evaluated for predicting transfusion needs, vasopressor duration, organ dysfunction, or ICU length of stay; the observed 24-hour nadir and 72-hour partial recovery provide practical time-anchors for sampling and longitudinal monitoring. Therapeutic implications aligned to molecular regulators and the microbiome (hypothesis-generating). Building directly on our MR signals and the staged microbiome shifts, we nominate phase-specific, mechanism-aligned strategies: (i) Barrier/HIF axis (protective HIF1A)—stabilize HIF signaling and improve mucosal perfusion in the acute 0–24 h window, combined with postbiotic short-chain fatty acids to preserve epithelial integrity and limit Proteobacteria expansion [68]; (ii) Cytoprotection/HSP axis (protective HSPB1)—pursue HSP co-induction together with prebiotic/probiotic approaches that preferentially expand SCFA-producing commensals (e.g., *Blautia*, *Romboutsia*) in 24–72 h; (iii) Apoptosis/APAF1 axis (risk)—test SCFA/postbiotic and tryptophan–AhR–modulating dietary interventions to attenuate epithelial apoptosis and inflammatory tone while the community transitions from the acute *Lactobacillus* bloom to a more complex configuration; and (iv) Coagulation/F7–F10 axis (risk)—evaluate microbiome strategies constraining Gram-negative pathobionts (e.g., rationally selected probiotics or bacteriophage candidates) alongside viscoelastic-guided hemostatic care. These proposals are strictly hypothesis-generating and require staged validation. Supplementary Fig. S4 aligns each regulator with target taxa, endpoints, and sampling windows (24 h/72 h) to guide trial design.

#### Rationale for not performing protein assays in this phase and the planned validation

The present study was purposefully conceived as a discovery-oriented integration of MR inference with time-resolved microbiome profiling to delineate robust ecological trajectories and generate a falsifiable “protein–microbiota–HS” regulatory axis. Given finite animal material and the need to power sequencing-based endpoints, we prioritized 16 S and LEfSe/PICRUSt2 analyses rather than tissue/plasma protein assays in this phase. To explicitly address this gap, we have prespecified a follow-up validation plan: (i) quantify heat shock protein beta-1 (HSPB1), hypoxia-inducible factor 1-alpha (HIF1A), apoptotic protease activating factor-1 (APAF1), coagulation factor VII (F7), and coagulation factor X (F10) in colon and ileum mucosa, liver, lung, and plasma at 0, 24, and 72 h using Western blot for tissue abundance, immunohistochemistry for spatial signals in gut, liver, and lung, and enzyme-linked immunosorbent assay for plasma; (ii) pair protein measurements with key genera and alpha/beta diversity indices; and (iii) model protein–microbiota coupling via linear mixed-effects and multivariate frameworks with multiple-testing control. Tissue prioritization and rationale: we will prioritize colon and ileum mucosa as the primary barrier under hypoperfusion to index epithelial hypoxia and stress responses (hypoxia-inducible factor 1-alpha and heat shock protein beta-1) and apoptosis (apoptotic protease activating factor-1); liver as the site of coagulation factor synthesis and regulation (coagulation factor VII and coagulation factor X); lung as a canonical remote-organ hypoxia and injury readout in hemorrhagic shock; and plasma as a clinically translatable matrix enabling serial measurement of circulating markers. This design directly tests MR-inferred directions and mechanistically anchors the microbiome shifts observed here.

#### Planned validation endpoints for microbiome-targeted hypotheses

We will pre-specify (a) co-primary protein endpoints (HSPB1, HIF1A, APAF1, F7, F10) and microbiome endpoints (alpha diversity; Bray–Curtis; *Lactobacillus*, *Blautia*, *Romboutsia*, Proteobacteria) under standard resuscitation versus no-resuscitation conditions; (b) prebiotic/probiotic and postbiotic butyrate arms to test effects on epithelial apoptosis markers and coagulation surrogates; and (c) mixed-effects modeling of protein–microbiome coupling to identify responders and optimal timing. In addition, to directly examine microbiome functional pathways beyond inference, we will incorporate shotgun metagenomics and metatranscriptomics together with targeted metabolomics focusing on short-chain fatty acids, amino acids, and bile acids at 0 h, 24 h, and 72 h in colon mucosa, ileum mucosa, feces, and plasma. To mechanistically disambiguate barrier protection versus systemic anti-inflammatory effects, we will additionally quantify tight-junction proteins (ZO-1 and occludin), mucin (MUC2), and epithelial apoptosis markers (cleaved caspase-3) in colon/ileum mucosa, and assess intestinal microthrombi (fibrin/platelet immunohistochemistry), mucosal perfusion/hypoxia proxies, and circulating inflammatory cytokines (e.g., TNF-α, IL-6) to directly link APAF1 activity and F7/F10-driven coagulation to microbiome shifts.

#### Why within-subject longitudinal sampling was not implemented in this phase

While sampling the same rats before hemorrhage and at 24/72 h would better control inter-individual variability, we prioritized an independent-cohort design to avoid (i) handling- and cage-change–induced microbiome shifts and carry-over effects between time points, (ii) confounding from post-hemorrhage care that would differentially affect subsequent samples in a within-subject scheme, and (iii) survivor bias and underpowering at 72 h. We therefore powered each time point with independent animals, implemented randomization and standardized sampling, balanced co-housing/cage distribution, and quantified dispersion with cage-stratified permutations. A dedicated within-subject longitudinal validation (baseline/24 h/72 h) is preplanned to directly measure intra-individual trajectories and cross-validate the present time-resolved signatures. Feasibility of a longitudinal design in follow-up work. In planned studies, we will track the same rats at baseline, 24 h, and 72 h using noninvasive fecal collection performed in the home cage to minimize handling-induced perturbations; anesthesia is not required. Hemorrhage induction, resuscitation care, housing, and diet will be standardized across time points. Statistical analysis will adopt linear mixed-effects models with a random intercept for rat and a fixed effect of time, including cage as a blocking factor. Sample-size calculations target 10–12 rats to achieve approximately 80% power under an anticipated within-animal correlation of 0.4–0.5. This design will directly quantify intra-individual trajectories and reduce the influence of inter-individual variability on effect-size estimates.

## Conclusion

This study systematically elucidated the causal roles of heat shock proteins, apoptotic regulators, hypoxia-inducible factors, and coagulation proteins in hemorrhagic shock (HS), while uncovering microbiota-mediated regulatory pathways that may underlie these associations. Using Mendelian randomization, we identified HSPB1 and HIF1A as protective genetic factors, whereas APAF1, F7, and F10 were linked to increased HS risk—revealing a bidirectional regulatory framework of stress-response proteins in HS pathogenesis. These findings are consistent with their known roles in coagulation pathways; however, coagulation activity was not directly assessed in this study and requires further experimental validation. Taken together, our findings support a working model in which HSPB1/HIF1A protect predominantly through intestinal mucosal barrier stabilization, whereas APAF1-driven apoptosis and F7/F10-mediated coagulation perturb gut ecology via barrier compromise and microvascular hypoperfusion, respectively.

In a rat model, HS led to substantial disruptions in gut microbial diversity and structure, including loss of beneficial taxa, expansion of opportunistic species, and temporally staged remodeling of metabolic pathways—most notably those involved in translation and amino acid metabolism. Microbial succession patterns closely mirrored the inferred protein-driven pathways at both phylogenetic and functional levels, suggesting that the gut microbiota may serve as a key intermediary in the regulation of HS.

Taken together, this study provides integrative evidence for MR-inferred protein–HS associations alongside microbiome restructuring. A protein–microbiota–HS regulatory axis is hypothesized but requires direct protein-level validation. These findings enrich our understanding of the pathophysiological landscape of hemorrhagic stress and highlight the therapeutic potential of microbiota-targeted interventions. In parallel, we predict that standard clinical resuscitation will moderate the dysbiosis observed here, a hypothesis we will test directly in a preregistered, parallel-group resuscitation arm. Additionally, genus-level trajectories identify Blautia (72 h increase) and Lactobacillus normalization as candidate stool biomarkers of recovery. We will prospectively test whether SCFA supplementation and probiotic interventions improve physiologic outcomes and shift these biomarkers in the favorable direction.

This study has several important limitations. First, our PICRUSt2-based functional readouts are predictive rather than directly measured; we did not perform metagenomics, metatranscriptomics, metaproteomics, or metabolomics, and therefore functional interpretations are hypothesis-generating. In subsequent work, we will evaluate pathway enrichment using shotgun metagenomics and metatranscriptomics and will examine metabolite outputs with targeted metabolomics focused on short-chain fatty acids, amino acids, and bile acids at 0 h, 24 h, and 72 h in colon mucosa, ileum mucosa, feces, and plasma. Second, we did not quantify protein expression of heat shock protein beta-1, hypoxia-inducible factor 1-alpha, apoptotic protease activating factor-1, and coagulation factors VII and X in rat tissues or plasma in this discovery phase, because animal material and resources were a priori allocated to time-resolved microbiome endpoints. We therefore explicitly pre-plan protein-level validation (Western blot/ELISA/IHC at 0/24/72 h in colon/ileum mucosa, liver, and lung) paired with microbial readouts and modeled via linear mixed-effects/multivariate approaches to directly test protein–microbiota coupling. Third, the species scope is limited to male Sprague–Dawley rats; potential sex differences and inter-species generalizability remain to be determined. Fourth, we lack external validation: MR estimates were not replicated in independent datasets, and the microbiome signatures were not tested in human HS cohorts. Fifth, this discovery-phase animal model intentionally omitted fluid and blood resuscitation to isolate the ecological effects of hypoperfusion. Therefore, generalizability to standard-of-care conditions is limited. We have preregistered follow-up studies including crystalloid and packed red blood cell resuscitation arms to directly test whether reduced gut hypoxia stabilizes microbiome composition and function. Additionally, because we used independent cohorts at each time point, the results may be influenced by between-animal variability; although we mitigated this with randomization, balanced co-housing, and dispersion analyses, this design precludes within-animal causal attribution and may have attenuated some effect sizes, thereby underscoring the need for a within-subject longitudinal follow-up.Finally, our GWAS datasets were derived predominantly from European populations, which may limit generalizability. Future studies should incorporate larger, multi-ethnic genetic resources, external replication, human validation cohorts, and interventional designs to more definitively assess clinical applicability. Future research should broaden the population scope and incorporate transcriptomic, proteomic, and interventional approaches to further clarify the microbe-mediated regulatory axis in HS.

Standard care for human hemorrhagic shock includes early fluid and blood resuscitation to restore perfusion and oxygen delivery. Reduced intestinal hypoxia is expected to stabilize epithelial barrier function and limit blooms of stress-responsive taxa (e.g., Proteobacteria), while promoting recovery of commensals and SCFA-associated lineages. Consistent with our MR findings (protective HSPB1/HIF1A), mitigating hypoxia and cellular stress should lessen selective pressures that drive acute community restructuring. We therefore predict that resuscitated animals will exhibit smaller declines in alpha diversity and attenuated beta-diversity separation versus non-resuscitated counterparts—a prospectively testable translational hypothesis embedded in our follow-up design. Accordingly, our resuscitation arm is designed to prospectively confirm these microbiome effects with predefined ecological endpoints and physiologic perfusion targets, providing a direct test of clinical relevance.

## Supplementary Information

Below is the link to the electronic supplementary material.

Supplementary Material 1

## Data Availability

The datasets used and analyzed during the current study are available from the corresponding author on reasonable request. The genomic data for Mendelian randomization analysis were sourced from publicly accessible databases, including the UK Biobank, as described in the Materials and Methods section.
