# Supplementary material for: Host stress proteins shape hemorrhagic shock via gut microbiota: evidence from Mendelian randomization and animal models
Source: J Transl Med. 2025 Nov 20;23:1324. doi: 10.1186/s12967-025-07364-8 (PMC12632137; doi:10.1186/s12967-025-07364-8)
Supplement: Supplementary file 1 — Supplementary Material 1 [file 12967_2025_7364_MOESM1_ESM.docx]

Fig.S1 Scatter and funnel plots of the apoptosis-related gene APAF1 in association with hemorrhagic shock


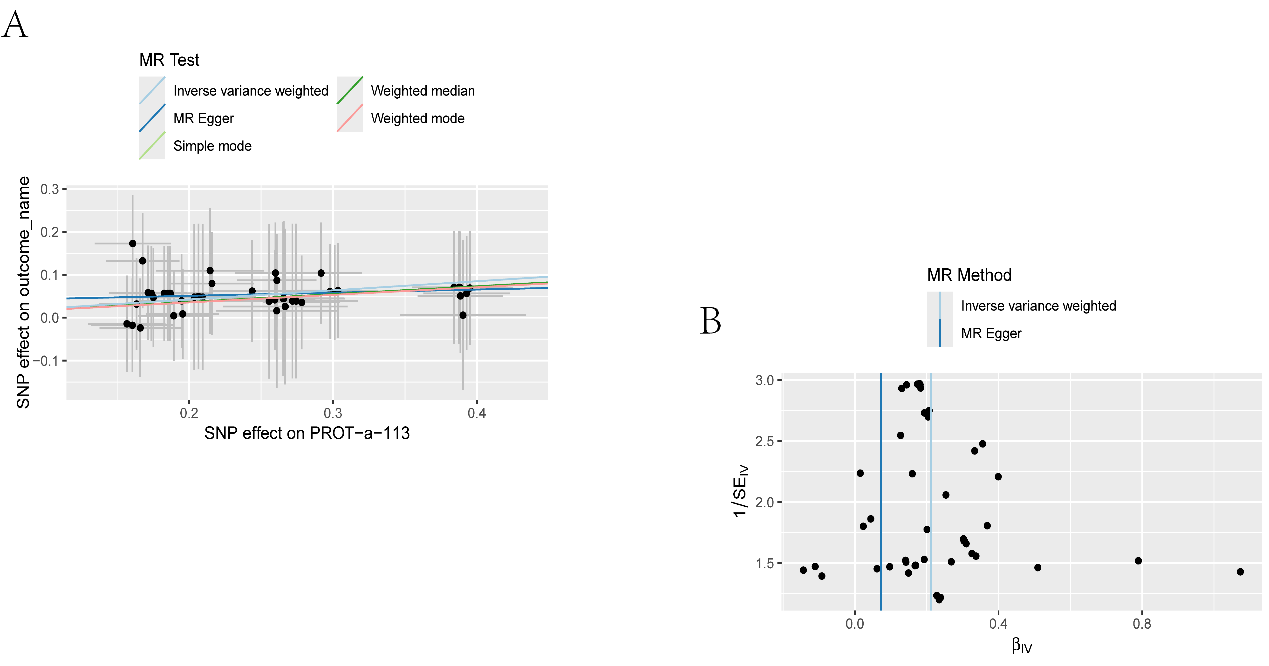


Fig. S2 Scatter and funnel plots of the hypoxia-inducible factor gene HIF1A in association with hemorrhagic shock


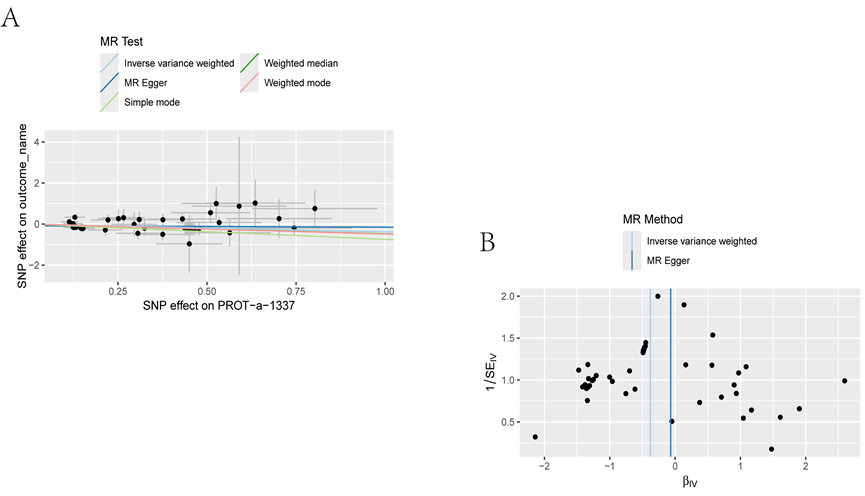


Fig. S3 Supplementary overview of sequencing data

(A) OTU petal plot — sample A

(B) OTU petal plot — sample B

(C) OTU petal plot — sample C


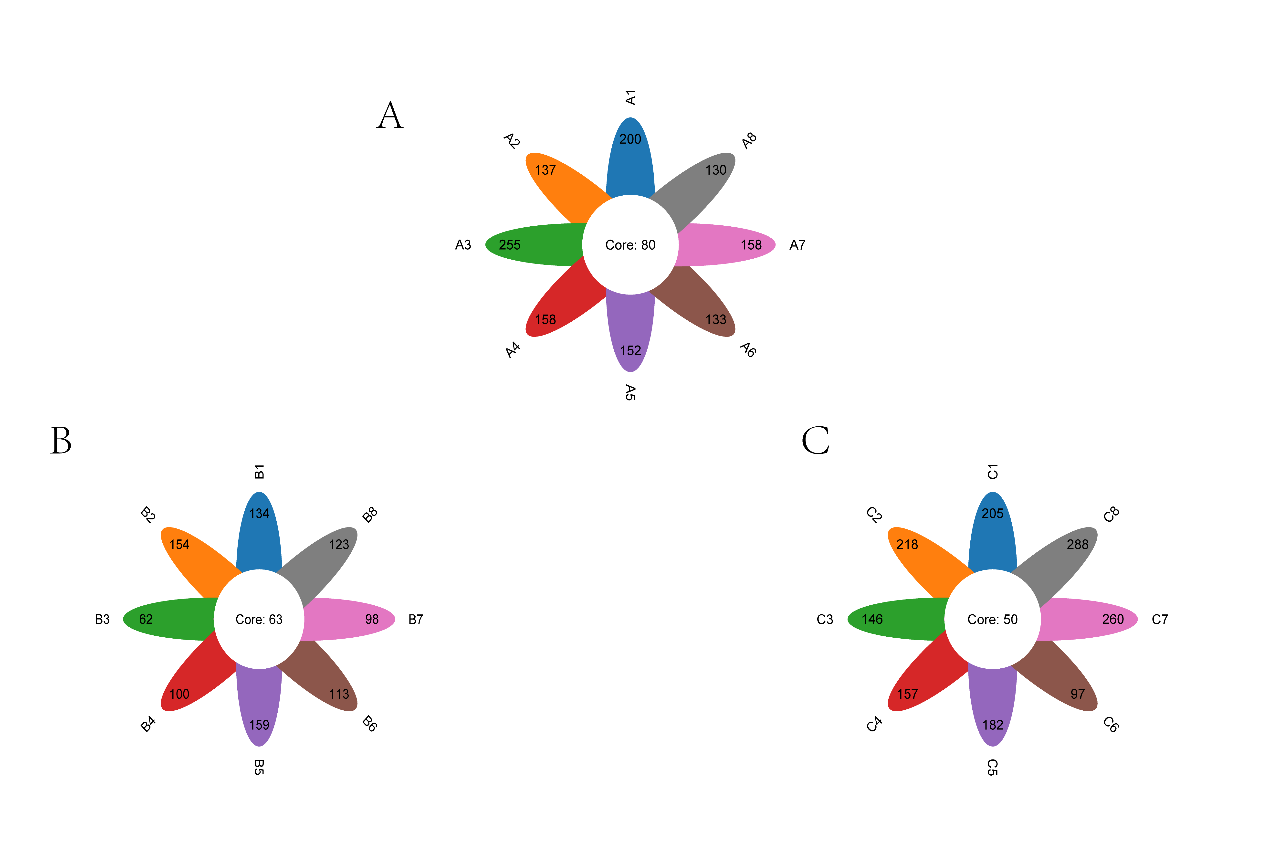


Fig. S4. Conceptual schematic of the protein–microbiota–hemorrhagic shock regulatory axis


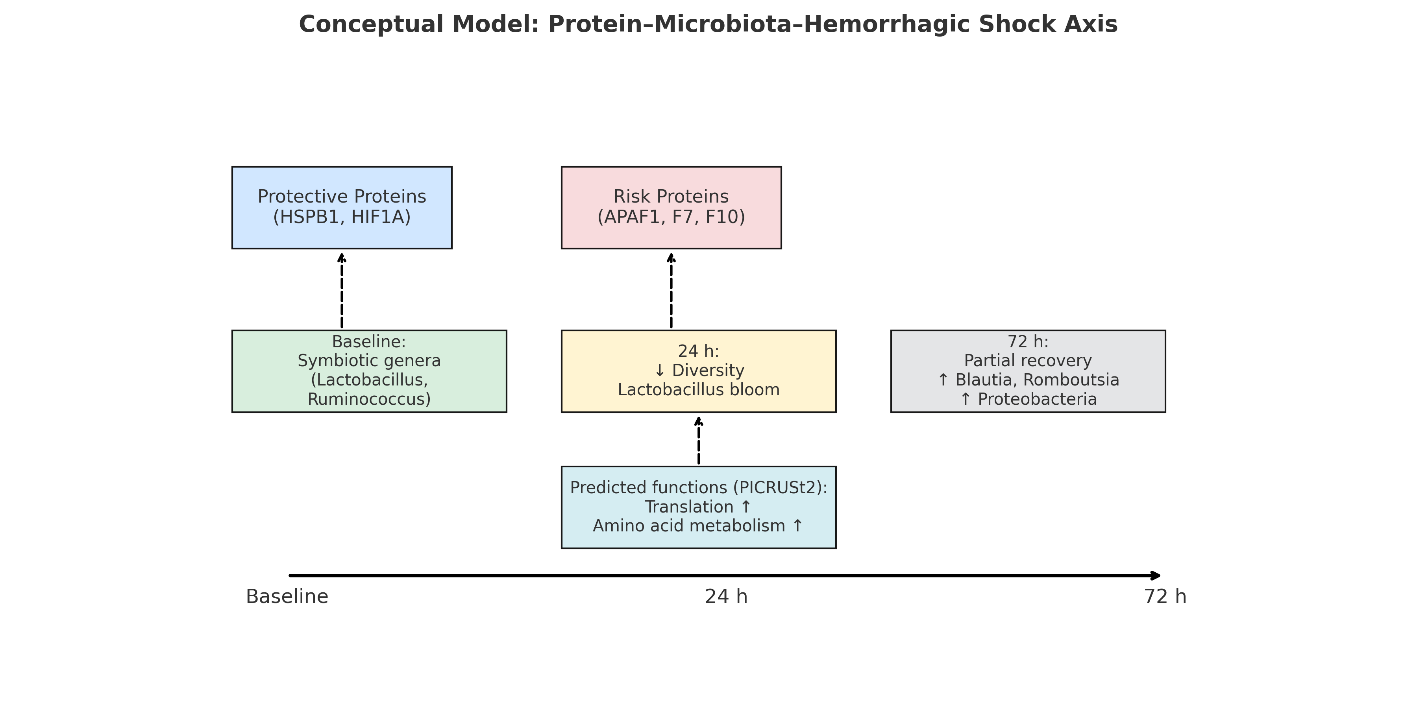


NOTE: The schematic summarizes the hypothesized regulatory axis linking host proteins, gut microbiota, and hemorrhagic shock progression. Protective proteins (HSPB1, HIF1A) and risk proteins (APAF1, F7, F10) were identified by MR analyses. Microbiome dynamics show a decline in alpha diversity with a transient bloom of Lactobacillus at 24 h, followed by partial recovery at 72 h with enrichment of Blautia and Romboutsia and an increase in Proteobacteria. Functional predictions from PICRUSt2 indicated enhanced translation and amino acid metabolism at 72 h. Solid arrows denote observed associations; dashed arrows denote hypothesized mediations. This model is conceptual and hypothesis-generating, requiring protein-level and clinical validation.

Table S1 Heat shock protein–related genes implicated in hemorrhagic shock

| Gene Symbol | Description | GC Id | Relevance score |
| --- | --- | --- | --- |
| HSPB1 | Heat Shock Protein Family B (Small) Member 1 | GC07P076302 | 52.485 |
| HSP90AA1 | Heat Shock Protein 90 Alpha Family Class A Member 1 | GC14M102080 | 51.4495 |
| HSPA1A | Heat Shock Protein Family A (Hsp70) Member 1A | GC06P181910 | 49.52698 |
| HSPA4 | Heat Shock Protein Family A (Hsp70) Member 4 | GC05P133132 | 49.51952 |
| HSPA8 | Heat Shock Protein Family A (Hsp70) Member 8 | GC11M123057 | 47.39293 |
| HSPA1B | Heat Shock Protein Family A (Hsp70) Member 1B | GC06P181909 | 37.07334 |
| HSP90AB1 | Heat Shock Protein 90 Alpha Family Class B Member 1 | GC06P044246 | 36.55441 |
| HSPD1 | Heat Shock Protein Family D (Hsp60) Member 1 | GC02M197486 | 36.34655 |
| HSPB2 | Heat Shock Protein Family B (Small) Member 2 | GC11P113461 | 34.59016 |
| HSPA5 | Heat Shock Protein Family A (Hsp70) Member 5 | GC09M125234 | 33.7479 |
| HSPB6 | Heat Shock Protein Family B (Small) Member 6 | GC19M108696 | 32.90313 |
| HSPA1L | Heat Shock Protein Family A (Hsp70) Member 1 Like | GC06M031809 | 32.35845 |
| HSPB8 | Heat Shock Protein Family B (Small) Member 8 | GC12P119173 | 32.22935 |
| HSPA9 | Heat Shock Protein Family A (Hsp70) Member 9 | GC05M138554 | 28.31383 |
| HSPA6 | Heat Shock Protein Family A (Hsp70) Member 6 | GC01P161524 | 27.60378 |
| HSPA14 | Heat Shock Protein Family A (Hsp70) Member 14 | GC10P014860 | 27.23714 |
| HSPB3 | Heat Shock Protein Family B (Small) Member 3 | GC05P055032 | 27.09047 |
| HSP90B1 | Heat Shock Protein 90 Beta Family Member 1 | GC12P103930 | 25.61634 |
| DNAJA2 | DnaJ Heat Shock Protein Family (Hsp40) Member A2 | GC16M046955 | 17.56425 |
| DNAJB11 | DnaJ Heat Shock Protein Family (Hsp40) Member B11 | GC03P186567 | 16.68058 |
| DNAJC9 | DnaJ Heat Shock Protein Family (Hsp40) Member C9 | GC10M073183 | 16.63341 |
| DNAJC6 | DnaJ Heat Shock Protein Family (Hsp40) Member C6 | GC01P065248 | 14.24872 |
| DNAJC21 | DnaJ Heat Shock Protein Family (Hsp40) Member C21 | GC05P034929 | 14.21525 |
| DNAJC3 | DnaJ Heat Shock Protein Family (Hsp40) Member C3 | GC13P095677 | 13.38792 |
| DNAJB12 | DnaJ Heat Shock Protein Family (Hsp40) Member B12 | GC10M072332 | 11.6898 |
| DNAJC17 | DnaJ Heat Shock Protein Family (Hsp40) Member C17 | GC15M040765 | 11.20579 |

Table S2 Apoptosis-related genes implicated in hemorrhagic shock

| Gene Symbol | Description | GC Id | Relevance score |
| --- | --- | --- | --- |
| APAF1 | Apoptotic Peptidase Activating Factor 1 | GC12P098645 | 7.767968 |
| ACIN1 | Apoptotic Chromatin Condensation Inducer 1 | GC14M023058 | 2.207055 |

Table S3 Hypoxia-inducible factor–related genes implicated in hemorrhagic shock

| Gene Symbol | Description | GC Id | Relevance score |
| --- | --- | --- | --- |
| HIF1A | Hypoxia Inducible Factor 1 Subunit Alpha | GC14P061695 | 13.61109 |
| EGLN1 | Egl-9 Family Hypoxia Inducible Factor 1 | GC01M231363 | 3.870906 |
| EGLN3 | Egl-9 Family Hypoxia Inducible Factor 3 | GC14M033924 | 3.608542 |
| HIF3A | Hypoxia Inducible Factor 3 Subunit Alpha | GC19P046297 | 2.016753 |

Table S4 Coagulation factor–related genes implicated in hemorrhagic shock

| Gene Symbol | Description | GC Id | Relevance score |
| --- | --- | --- | --- |
| F2 | Coagulation Factor II, Thrombin | GC11P049059 | 31.43073 |
| F5 | Coagulation Factor V | GC01M169511 | 28.06898 |
| F8 | Coagulation Factor VIII | GC0XM154835 | 26.41957 |
| F3 | Coagulation Factor III, Tissue Factor | GC01M095040 | 24.93286 |
| F10 | Coagulation Factor X | GC13P113122 | 23.91101 |
| F7 | Coagulation Factor VII | GC13P113105 | 22.71577 |
| F9 | Coagulation Factor IX | GC0XP139530 | 20.49468 |
| F13A1 | Coagulation Factor XIII A Chain | GC06M006144 | 18.98725 |
| PROC | Protein C, Inactivator Of Coagulation Factors Va And VIIIa | GC02P127418 | 11.35884 |
| F12 | Coagulation Factor XII | GC05M177402 | 10.28654 |
| F13B | Coagulation Factor XIII B Chain | GC01M197008 | 10.1178 |
| MCFD2 | Multiple Coagulation Factor Deficiency 2, ER Cargo Receptor Complex Subunit | GC02M046901 | 8.659295 |
| F2R | Coagulation Factor II Thrombin Receptor | GC05P076716 | 7.686877 |
| F8A1 | Coagulation Factor VIII Associated 1 | GC0XP154886 | 2.973739 |

**Table S5 Summary statistics and quality metrics of sequencing data for each sample**

| Sample ID | Raw Reads | Clean Reads | Denoised Reads | Merged Reads | Non-chimeric Reads |
| --- | --- | --- | --- | --- | --- |
| A1 | 79989 | 72359 | 72208 | 70426 | 67451 |
| A2 | 79965 | 71923 | 71800 | 69952 | 63556 |
| A3 | 79895 | 72813 | 72566 | 69400 | 59482 |
| A4 | 79970 | 73406 | 73194 | 71017 | 64437 |
| A5 | 79978 | 72615 | 72427 | 70598 | 67302 |
| A6 | 80149 | 73865 | 73676 | 70317 | 63509 |
| A7 | 80071 | 74248 | 74115 | 72026 | 67557 |
| A8 | 79968 | 72871 | 72675 | 70931 | 64550 |
| B1 | 79912 | 72805 | 72649 | 70558 | 62970 |
| B2 | 79944 | 72716 | 72616 | 71405 | 65759 |
| B3 | 69042 | 60827 | 60791 | 60021 | 57665 |
| B4 | 79926 | 72143 | 72036 | 71022 | 64935 |
| B5 | 80220 | 73127 | 72999 | 71382 | 65383 |
| B6 | 71811 | 64059 | 63993 | 62994 | 56965 |
| B7 | 79990 | 71205 | 71114 | 70237 | 64786 |
| B8 | 80124 | 73416 | 73279 | 71788 | 64652 |
| C1 | 79870 | 73043 | 72857 | 70187 | 65830 |
| C2 | 79995 | 75219 | 75007 | 71865 | 66209 |
| C3 | 79789 | 73321 | 73170 | 70426 | 67847 |
| C4 | 80017 | 73906 | 73779 | 72027 | 62754 |
| C5 | 62422 | 57974 | 57778 | 54537 | 50948 |
| C6 | 79813 | 72445 | 72344 | 70047 | 67022 |
| C7 | 80241 | 73328 | 73095 | 69395 | 62412 |
| C8 | 80060 | 73605 | 73385 | 69269 | 60443 |

Note:

Sample ID — sample identifier;

Raw Reads — total number of reads generated from initial sequencing;

Clean Reads — high-quality reads retained after quality control;

Denoised Reads — reads filtered to remove sequencing noise from Clean Reads;

Merged Reads — sequences obtained by merging overlapping Denoised Reads;

Non-chimeric Reads — final number of valid reads after chimera removal.

**Table S6 Alpha diversity metrics derived from sequencing data**

| Sample ID | ACE | Chao1 | Simpson | Shannon | PD_whole_tree | Coverage |
| --- | --- | --- | --- | --- | --- | --- |
| A1 | 470.5995 | 471.125 | 0.8642 | 5.3244 | 18.5667 | 0.9998 |
| A2 | 381.0167 | 384.1111 | 0.8948 | 5.0946 | 17.6882 | 0.9998 |
| A3 | 517.8874 | 517.3158 | 0.9449 | 5.7665 | 17.816 | 0.9999 |
| A4 | 433.4274 | 436 | 0.9458 | 5.7322 | 19.0876 | 0.9998 |
| A5 | 423.7793 | 429.1 | 0.916 | 5.5543 | 18.4167 | 0.9998 |
| A6 | 415.271 | 419.1765 | 0.9687 | 6.2345 | 18.5843 | 0.9997 |
| A7 | 426.4311 | 428.0667 | 0.9406 | 5.7235 | 19.1198 | 0.9998 |
| A8 | 372.3585 | 372.5714 | 0.8585 | 4.8334 | 17.3898 | 0.9999 |
| B1 | 375.4709 | 379.8 | 0.8867 | 4.9838 | 18.1731 | 0.9998 |
| B2 | 363.8612 | 374.75 | 0.8238 | 4.3564 | 18.0998 | 0.9998 |
| B3 | 264.0912 | 267.4286 | 0.8343 | 4.2906 | 16.3426 | 0.9998 |
| B4 | 315.944 | 316.1538 | 0.8266 | 4.0324 | 17.0732 | 0.9999 |
| B5 | 382.3089 | 396 | 0.8633 | 4.736 | 17.8731 | 0.9997 |
| B6 | 311.995 | 314.6 | 0.8068 | 3.8373 | 16.5041 | 0.9998 |
| B7 | 303.6108 | 305.0588 | 0.8236 | 3.9903 | 16.5229 | 0.9998 |
| B8 | 353.2476 | 355.5 | 0.8557 | 4.6517 | 17.3009 | 0.9998 |
| C1 | 483.0428 | 487.3333 | 0.9677 | 6.3049 | 17.9772 | 0.9998 |
| C2 | 516.1777 | 521.5455 | 0.9713 | 6.4484 | 18.6592 | 0.9998 |
| C3 | 457.1646 | 457.5882 | 0.9691 | 6.431 | 20.333 | 0.9998 |
| C4 | 350.6124 | 350.4 | 0.9082 | 4.6693 | 15.684 | 0.9999 |
| C5 | 491.1222 | 492.2727 | 0.9818 | 6.8203 | 19.1711 | 0.9998 |
| C6 | 363.3472 | 368.2 | 0.9568 | 6.0772 | 17.1463 | 0.9999 |
| C7 | 565.0931 | 569.8 | 0.9767 | 6.6884 | 19.5074 | 0.9998 |
| C8 | 557.3719 | 561.0909 | 0.9797 | 6.5895 | 18.8918 | 0.9998 |

Note:

ACE – Abundance-based Coverage Estimator; estimates community richness based on species abundance.

Chao1 – A widely used non-parametric estimator of species richness.

Simpson – An index of microbial diversity that accounts for both species richness and evenness.

Shannon – Shannon-Wiener index; quantifies microbial diversity based on species abundance and distribution.

PD_whole_tree – Phylogenetic diversity derived from the entire evolutionary tree; higher values indicate greater microbial community complexity.

Coverage – Sequencing library coverage per sample, reflecting sampling completeness.
